# Multimodal data integration for predicting progression risk in castration-resistant prostate cancer using deep learning: a multicenter retrospective study

**DOI:** 10.3389/fonc.2024.1287995

**Published:** 2024-03-14

**Authors:** Chuan Zhou, Yun-Feng Zhang, Sheng Guo, Yu-Qian Huang, Xiao-Ni Qiao, Rong Wang, Lian-Ping Zhao, De-Hui Chang, Li-Ming Zhao, Ming-Xu Da, Feng-Hai Zhou

**Affiliations:** ^1^ The First Clinical Medical College of Lanzhou University, Lanzhou, China; ^2^ National Health Commission of People’s Republic of China (NHC) Key Laboratory of Diagnosis and Therapy of Gastrointestinal Tumor, Gansu Provincial Hospital, Lanzhou, China; ^3^ The First Clinical Medical College of Gansu University of Chinese Medicine, Lanzhou, China; ^4^ Department of Center of Medical Cosmetology, Chengdu Second People’s Hospital, Chengdu, China; ^5^ Department of Urology, The 940 Hospital of Joint Logistics Support Force of Chinese PLA, Lanzhou, China; ^6^ Department of Radiology, Gansu Provincial Hospital, Lanzhou, China; ^7^ Department of Urology, Second People’s Hospital of Gansu Province, Lanzhou, China; ^8^ Department of Urology, Gansu Provincial Hospital, Lanzhou, China

**Keywords:** radiomics, pathomics, castration-resistant prostate cancer, deep learning, multi-modal

## Abstract

**Purpose:**

Patients with advanced prostate cancer (PCa) often develop castration-resistant PCa (CRPC) with poor prognosis. Prognostic information obtained from multiparametric magnetic resonance imaging (mpMRI) and histopathology specimens can be effectively utilized through artificial intelligence (AI) techniques. The objective of this study is to construct an AI-based CRPC progress prediction model by integrating multimodal data.

**Methods and materials:**

Data from 399 patients diagnosed with PCa at three medical centers between January 2018 and January 2021 were collected retrospectively. We delineated regions of interest (ROIs) from 3 MRI sequences viz, T2WI, DWI, and ADC and utilized a cropping tool to extract the largest section of each ROI. We selected representative pathological hematoxylin and eosin (H&E) slides for deep-learning model training. A joint combined model nomogram was constructed. ROC curves and calibration curves were plotted to assess the predictive performance and goodness of fit of the model. We generated decision curve analysis (DCA) curves and Kaplan–Meier (KM) survival curves to evaluate the clinical net benefit of the model and its association with progression-free survival (PFS).

**Results:**

The AUC of the machine learning (ML) model was 0.755. The best deep learning (DL) model for radiomics and pathomics was the ResNet-50 model, with an AUC of 0.768 and 0.752, respectively. The nomogram graph showed that DL model contributed the most, and the AUC for the combined model was 0.86. The calibration curves and DCA indicate that the combined model had a good calibration ability and net clinical benefit. The KM curve indicated that the model integrating multimodal data can guide patient prognosis and management strategies.

**Conclusion:**

The integration of multimodal data effectively improves the prediction of risk for the progression of PCa to CRPC.

## 1 Introduction

Prostate cancer (PCa) affects men worldwide and is a significant health concern, with a global incidence rate of 13.5% (1). Additionally, the mortality rate of 6.7% makes PCa the fifth leading cause of death among men (2). Androgen deprivation therapy (ADT) is considered the primary treatment modality for men diagnosed with advanced symptomatic PCa, also known as castration-sensitive PCa (CSPC) (3). However, subsequent to the initial favorable treatment response, it is frequently observed in PCa patients that there is a decline in response and eventual progression to CRPC, which is characterized by a dismal prognosis (3). The median duration and mean survival period of patients until progression to CRPC range from 18 to 24 months and 24 to 30 months (4, 5), respectively. The status of the depot condition (testosterone [TST] 50 ng/dL or 1.7 nmol/L) and subsequent disease development (a sustained rise in prostate-specific antigen [PSA] and progression seen in images) are now the two most important criteria for detecting CRPC. However, tailored precision medicine is limited by the use of monomodal indicators such as PSA and serum testosterone (6, 7). The early detection of CRPC can help physicians determine the optimal timing for administering second-line therapies, possibly increasing the survival rate among patients. Predicting the risk of CRPC is an important factor affecting prognosis in patients with severe PCa. There is an urgent need for early diagnosis and precise management of CRPC.

Despite advancements in technology, there are still persistent challenges in accurately detecting, characterizing, and monitoring cancers (8). The assessment of diseases through radiographic methods primarily relies on visual evaluations, which can be enhanced by advanced computational analyses. Notably, AI holds the potential to significantly improve the qualitative interpretation of cancer imaging by expert clinicians (9). This includes the ability to accurately delineate tumor volumes over time, infer the tumor’s genotype and biological progression from its radiographic phenotype, and predict clinical outcomes (10). Radiomics, and pathomics have rapidly emerged as cutting-edge techniques to aid and enhance the interpretation of vast medical imaging data, which may benefit clinical applications. The techniques have the ability to directly process images, giving rise to numerous subdomains for further research (11). Clinical outcomes, such as survival, response to treatment, and recurrence, may be accurately predicted using AI models that use multimodal data (12–14). The utilization of radiomics and pathomics exhibits significant promise in enhancing clinical decision-making processes and ultimately enhancing patient outcomes via medical imaging techniques (15–17).

Hence, to effectively and precisely anticipate the likelihood of developing CRPC without invasive procedures. We constructed radiomics and pathomics prediction models based on deep-learning algorithms and investigated their application value in clinical decision-making and the prognosis of PCa. This may allow more accurate prediction of the risk of CRPC and provide a reference for accurate diagnosis and treatment of PCa.

## 2 Materials and methods

Clinicopathological data from patients with PCa were acquired retrospectively from the electronic medical record system of the three centers (center A; center B; center C) after receiving approval from the ethics committee of the local institution. This retrospective study was also approved by the Ethics Committee of the Gansu Provincial Geriatrics Association (2022-61), and the requirement for informed consent was waived. Our research program was designed based on the AI model of a local institution.

### 2.1 Participants

We conducted a retrospective study including patients with a pathologically confirmed diagnosis of PCa from the three centers between January 2018 and February 2021. The inclusion criteria were (a) first pathological diagnosis of PCa; (b) use of the same ADT treatment regimen; (c) availability of all MRI scans within 30 days of PCa diagnosis to exclude confounding effects of medication on measurements; and (d) no missing stained tissue slides. The exclusion criteria were (a) missing clinical information; (b) poor quality of MRI images (inability to identify the specific location of the lesion); (c) poor quality of stained tissue slides (uneven staining); and (d) missing follow-up information.

**Figure 1:**
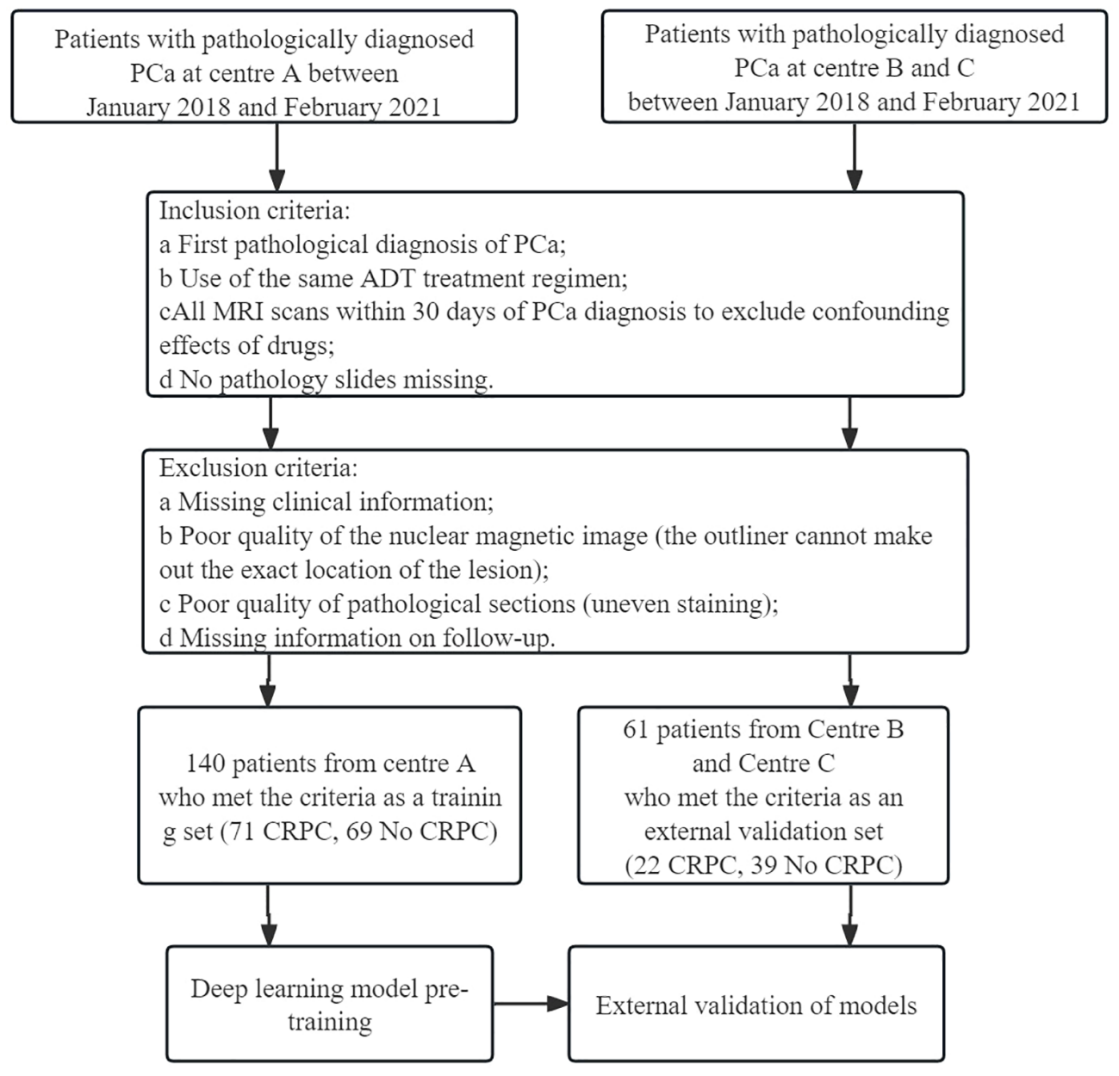
Flow chart of patient recruitment. Center **(A)** Gansu Provincial Hospital; Center **(B)** The 940 Hospital of Joint Logistics Support Force of Chinese PLA; Center **(C)** Second People’s Hospital of Gansu Province.

Clinical data from 399 patients with PCa were collected, including 254 from the Gansu Provincial Hospital (Center A), 112 from the 940 Hospital of Joint Logistics Support Force of Chinese PLA (Center B), and 33 from the Second People’s Hospital of Gansu Province (Center C). **Figure 1** shows the flowchart for patient recruitment.

### 2.2 Prostate tumor segmentation

A radiologist (R.W) with 5 years of experience in prostate MRI diagnosis and a urologist (FH.Z) with 30 years of experience in PCa MRI diagnosis were involved in delineating the regions of interest (ROIs). Disagreements regarding individual lesions were resolved after consultation with a third radiologist (LP. Z), and a consensus was attained. The radiologist were unaware of the patients’ CRPC status and adhered to the guidelines outlined the Prostate Imaging Reporting and Data System Version 2 (PI-RADS-V2). Once the delineation of the Region of Interest (ROI) was finalized, a random screening of the 11 features extracted from the ADC sequences was performed. Subsequently, Mann-Whitney U tests were conducted on both sets of features to ascertain the presence of any potential bias in the results obtained by the two experts (R.W and FH.Z) during the delineation process. The main sequence parameters of mp-MRI in **Supplementary Table 1**. The ITK-SNAP software, version 4.0.0 (http://itk-snap.org), was used to annotate the ROIs for each patient from three sequences, including T2-weighted (T2WI), diffusion-weighted imaging (DWI), and apparent diffusion coefficient (ADC). The volume of interest was created by overlapping the ROIs of each patient. To pretrain the DL model, 2-dimensional (2D) ROIs were extracted from the original images of the three sequences by using a clipping tool based on the tumor’s 3D segmentation mask. The standard protocol of Digital Imaging and Communications in Medicine (DICOM) is commonly used for managing medical imaging information and related data. To ensure data quality, we standardized it to a resampling format with a resolution of 1 cm × 1 cm × 1 cm and performed N4 bias correction on all images before delineation.

A pathologist (X.Z) selected a histopathological hematoxylin and eosin (H&E) slide (20×10 magnification) of a typical tumor area as the pathological image for the patient. To prevent data heterogeneity, we used Photoshop to adjust each histopathological slide to the same pixel size (640×480) for pretraining the DL model. Overall, 141 patients from Center A were included in the training group, while 60 patients from Center B and Center C were included in the external validation group for building ML and DL models.

### 2.3 Signature construction

#### 2.3.1 Radiomics signature construction

PyRadiomics (http://www.radiomics.io/pyradiomics.html) was used for extracting radiomics features. Additionally, the Z-score was employed for dataset standardization ([column−mean]/standard). The method involved using the Spearman correlation coefficient to evaluate the consistency among observers in feature extraction. Features with a correlation coefficient greater than 0.9 were considered reliable and formed a feature set for subsequent analysis. Normalization was performed by subtracting the mean value of each feature and dividing it by the standard deviation. The least absolute shrinkage and selection operator (LASSO) algorithm was used for feature selection and construction, with multiple iterations to assess the importance of each feature. Lastly, ML classifiers, such as logistic regression (LR) and support vector machines (SVM), were utilized to build the predictive models.

#### 2.3.2 DL signature construction

In this study, ResNet-50, ResNet-34, ResNet-18, Vgg19, and other deep transfer learning (DTL) models were used for model pretraining. The number of iterations (epochs) was set to 100, with a batch size of 32. Imagenet was employed as the regularization method. To enhance the interpretability of the model’s decision-making process, we applied the Gradient-weighted Class Activation Mapping (Grad-CAM) method for visual analysis of the model. This method utilizes the gradient information from the last convolutional layer of the neural network to generate a weighted fusion of the class activation map. This class activation map highlights the important regions of the classified target image, thereby allowing us to better understand the decision-making principles of the model.

#### 2.3.3 Construction of nomogram

We integrated radiomics models, DL models, and pathomics models to construct a nomogram and investigated the contributions of various modalities in the joint model.

### 2.4 Model evaluation

To evaluate the predictive performance of the models, we plotted ROC curves for each model and calculated the area under the curve (AUC) values. Decision curve analysis (DCA) curves and calibration curves were used to assess the net clinical benefit and goodness of fit of the joint model. Kaplan–Meier (KM) curves were used to evaluate its relationship with progression-free survival (PFS).

### 2.5 Statistical analysis

Statistical Package for Social Sciences (SPSS) 23.0 and R statistical software (version 3.6.1 R, https://www.r-project.org/) were used for statistical analysis. The Kolmogorov–Smirnov test was used to evaluate the normality of the measures, and those that conformed to a normal distribution were expressed as x ± s. The measures that did not conform to a normal distribution were expressed as the median (upper and lower quartiles). An independent samples t-test (normally distributed with equal variance) or Mann–Whitney U-test (skewed distribution or unequal variance) was used to compare the measures. Multi-factor LR analysis was used to screen out the independent predictors to construct the prediction model and plot the nomogram. The AUC of the receiver operating characteristics (ROC) was calculated to evaluate the discriminative power of the model. A DCA curve was plotted to compare the clinical value of the model. A p-value of <0.05 indicated a statistically significant difference.

## 3 Results

### 3.1 Clinical characteristics

**Figure 2:**
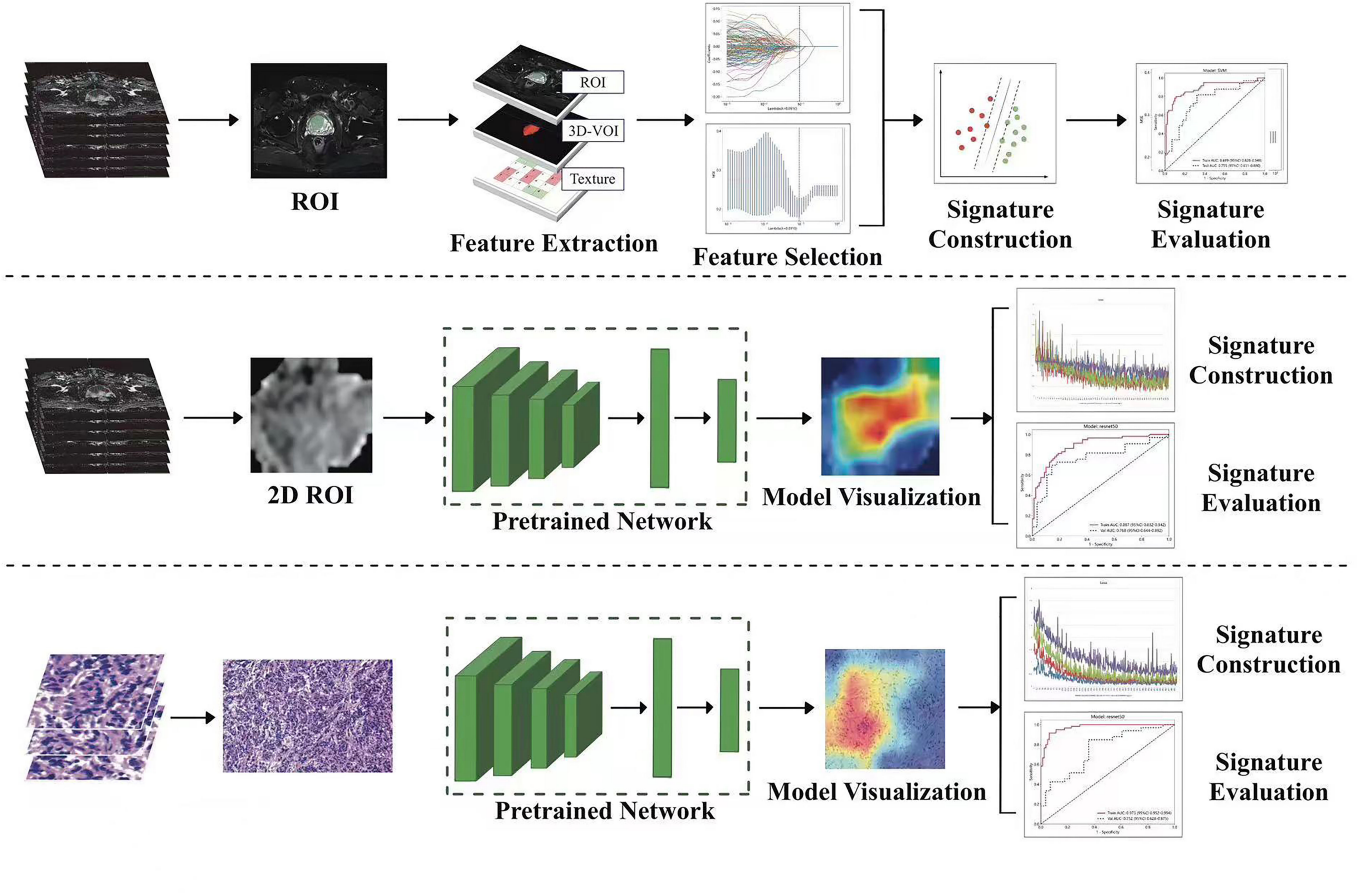
Schematic outline of the study. SVM, support vector machine; ROI, region of interest.

The study flow is shown in **Figure 2**. A total of 198 patients were excluded for not meeting the inclusion criteria, and 201 patients were included; 93 included patients progressed to CRPC. Statistical analysis revealed no significant differences in clinical features between the training and validation groups (**Table 1**).

**Table 1:** Comparison of clinical data of patients with prostate cancer in the training set and validation set.

Characteristic	Training Set (n=140)	External Validation Set (n=61)	t/Z/X^2^ Value	*P* Value
Age			0.000^c^	0.985
≤65	30	13		
>65	110	48		
BMI			0.563^c^	0.755
<25	96	40		
25-30	40	20		
>30	4	1		
BM			0.394^c^	0.530
yes	71	28		
no	69	33		
Gleason Score			0.915^c^	0.822
≤6	4	3		
3+4	15	7		
4+3	13	4		
≥8	108	47		
tPSA	61.99(31.49,100.00)	55.11(29.88,100.00)	-0.357^b^	0.721
Volume	46.30(32.13,67.18)	40.30(29.65,62.99)	-0.939^b^	0.348
PASD	1.11(0.59,1.94)	1.23(0.45,2.05)	-0.302^b^	0.763
ALP	84.00(64.25,130.75)	94.00(70.00,129.00)	-1.002^b^	0.316
Fbg	3.44(2.82,4.36)	3.37(2.88,4.35)	-0.514^b^	0.607
NEUT	3.57(2.91,4.82)	3.57(2.70,4.95)	-0.444^b^	0.657
Lym	1.36(0.93,1.85)	1.38(1.02,1.95)	-0.866^b^	0.386
M	0.44(0.35,0.56)	0.44(0.36,0.57)	-0.381^b^	0.703
Hb	142.00(126.25,154.00)	141.00(125.50,152.00)	-0.499^b^	0.618
PLT	173.50(137.00,215.25)	170.00(144.50,211.00)	-0.070^b^	0.944
SII	753.01±784.31	592.98±480.61	-1.769^a^	0.079
TST	11.20(1.13,18.98)	1.50(1.00,14.75)	-1.737^b^	0.082

a:statistical analysis performed using T-test;b:statistical analysis performed using Mann-Whitney test.c: statistical analysis performed using X^2^ test. BMI, Body Mass Index; BM, Bone Metastasis; PSAD, PSA density; ALP, Alkaline phosphatase; Fbg, Fibrinogen; NEUT, Neutrophil; Lym, lymphocyte; M, Monocyte; Hb, Hemoglobin; PLT, Platelet; SII, Systemic immune inflammatory index, SII= PLT* NLR; TST, testosterone.

### 3.2 Feature selection and signature construction

**Figure 3:**
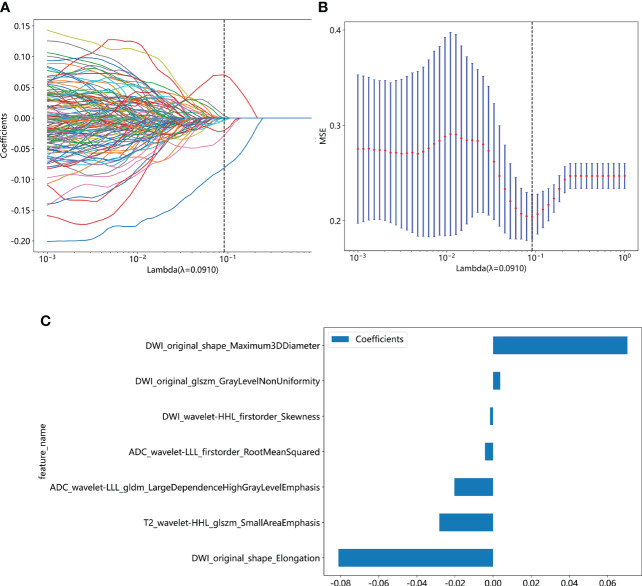
**(A)** Coefficient profiles of the features in the LASSO model are shown. Each feature is represented by a different color line indicating its corresponding coefficient. **(B)** Tuning parameter (λ) selection in the LASSO model. **(C)** Weights for each feature in the model. LASSO, least absolute shrinkage and selection operator.

**Figure 4:**
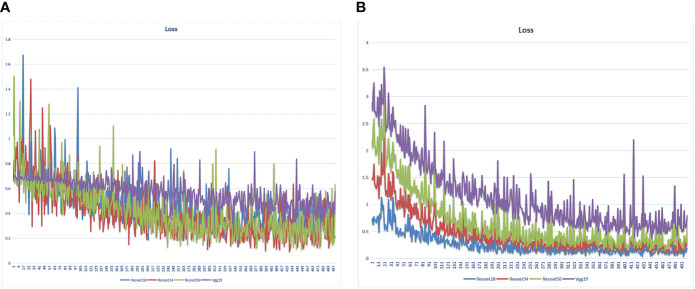
Loss value of different DL models in the training set varied with the iteration steps. **(A)** radiomics model **(B)** pathomics model.

**Figure 5:**
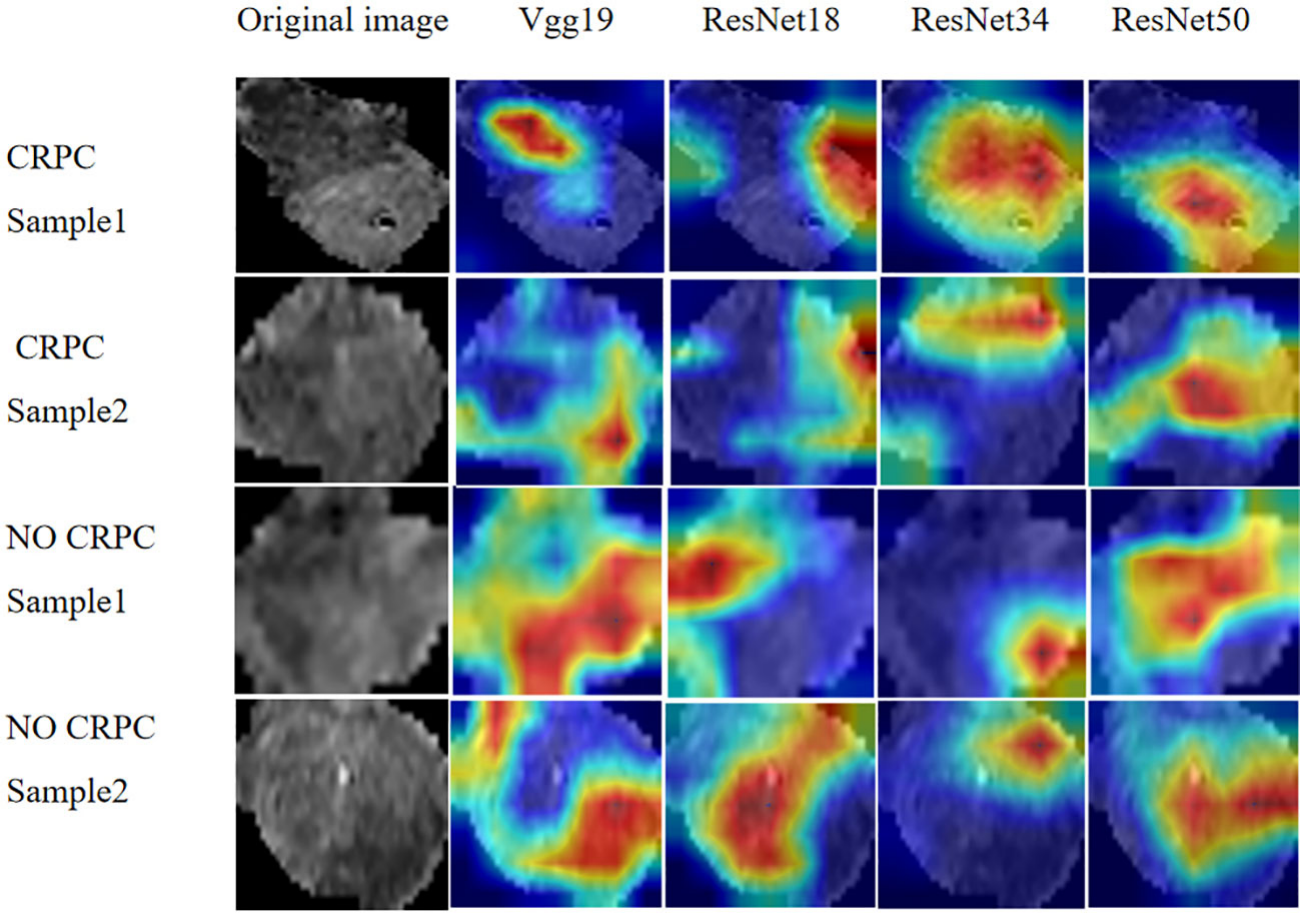
Regions of attention in prostate cancer MRI analysis with different DL models. MRI, magnetic resonance imaging.

We extracted 2553 radiomic features using PyRadiomics. According to the ROI results presented by the two experts, a random selection of 11 features derived from ADC sequences was subjected to a Mann-Whitney U test. The analysis revealed no statistically significant distinction between the two groups of features (**Supplementary Table 2**). Seven radiomic features were selected using the LASSO algorithm (**Figures 3A–C**). Three 2D ROIs with maximum cross-sections were chosen, and different deep-learning models were used for pretraining and external validation. Model evaluation (**Table 2**) demonstrated that ResNet-50 had better overall performance in the external validation set, with the lowest loss value. This indicates that ResNet-50 had fewer errors during the training process and converged faster than any other Convolutional Neural Network(CNN)model (**Figures 4A, B**). In terms of model interpretability, each model had distinct attention regions in the samples. In comparison, ResNet-50 had clearer attention regions primarily focused on the internal regions of the tumor, while the tumor regions in the surrounding tissue were not activated (**Figure 5**). Furthermore, the ResNet-50 model performed better in the ADC sequence among the three sequences (**Table 3**).

**Table 2:** Performance of different DL models.

	ModelName	Acc	AUC	95%CI	Sensitivity	Specificity	PPV	NPV	Precision	Recall	F1	Threshold	Cohort
0	resnet18	0.572	0.643	0.5514-0.7339	0.983	0.226	0.500	0.955	0.500	0.983	0.663	0.154	Train
1	resnet18	0.770	0.810	0.6693-0.9198	0.879	0.643	0.744	0.818	0.744	0.879	0.806	0.471	Test
2	resnet34	0.935	0.978	0.9584-0.9976	0.949	0.929	0.903	0.961	0.903	0.949	0.926	0.465	Train
3	resnet34	0.689	0.698	0.5663-0.8298	0.455	0.964	0.937	0.600	0.937	0.455	0.612	0.502	Test
4	resnet50	0.804	0.887	0.8318-0.9423	0.864	0.759	0.729	0.882	0.729	0.864	0.791	0.382	Train
5	resnet50	0.770	0.768	0.6436-0.8921	0.697	0.857	0.852	0.706	0.852	0.697	0.767	0.515	Test
6	vgg19	0.681	0.709	0.6213-0.7962	0.610	0.734	0.632	0.716	0.632	0.610	0.621	0.444	Train
7	vgg19	0.754	0.728	0.5948-0.8608	0.909	0.751	0.714	0.842	0.714	0.909	0.800	0.508	Test

**Table 3:** Performance of ResNet-50 in different sequences.

Sequence	Acc	AUC	95%CI	Sensitivity	Specificity	PPV	NPV	Precision	Recall	F1	Thres-hold	Cohort
T2	0.843	0.921	0.878-0.963	0.867	0.825	0.788	0.892	0.788	0.867	0.825	0.405	Train
	0.738	0.684	0.545-0.823	0.758	0.714	0.758	0.714	0.758	0.758	0.758	0.386	Test
DWI	0.686	0.710	0.620-0.799	0.741	0.646	0.606	0.773	0.606	0.741	0.667	0.368	Train
	0.689	0.714	0.584-0.845	0.727	0.643	0.706	0.667	0.706	0.727	0.716	0.534	Test
ADC	0.804	0.887	0.832-0.942	0.864	0.759	0.729	0.882	0.729	0.864	0.791	0.382	Train
	0.770	0.768	0.644-0.892	0.697	0.857	0.852	0.706	0.852	0.697	0.767	0.515	Test

### 3.3 Validation of radiomics and pathomics signature

**Figure 6:**
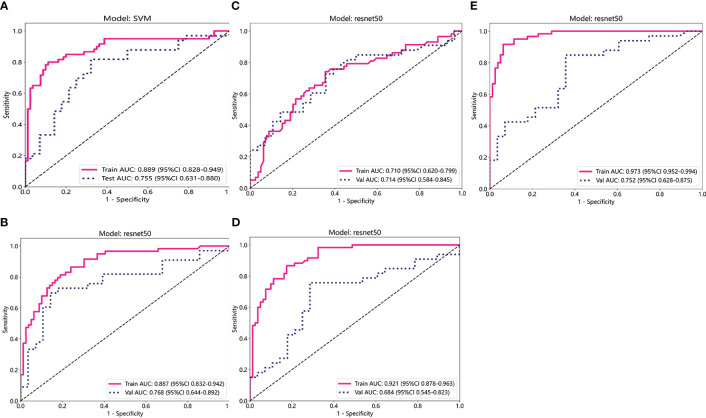
ROC curve analysis for each model. **(A)** Radiomics. **(B-D)** DL (ADC, DWI, and T2WI) **(E)** Pathomics. T2WI, T2-weighted imaging; DWI, diffusion-weighted imaging; ADC, apparent diffusion coefficient images; ROC, receiver operating characteristic.

**Figure 7:**
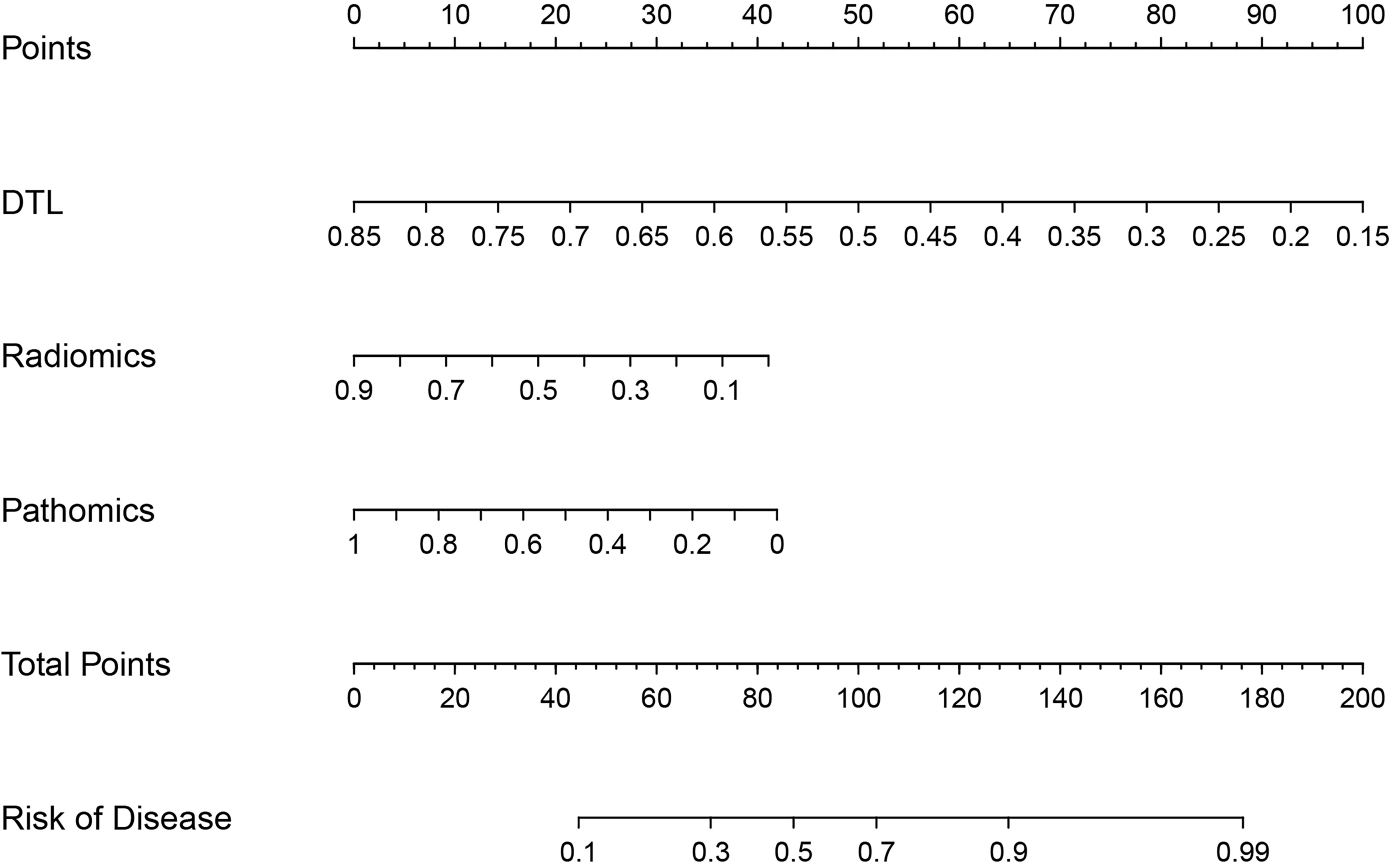
Nomodiagram of the combined model.

**Figure 8:**
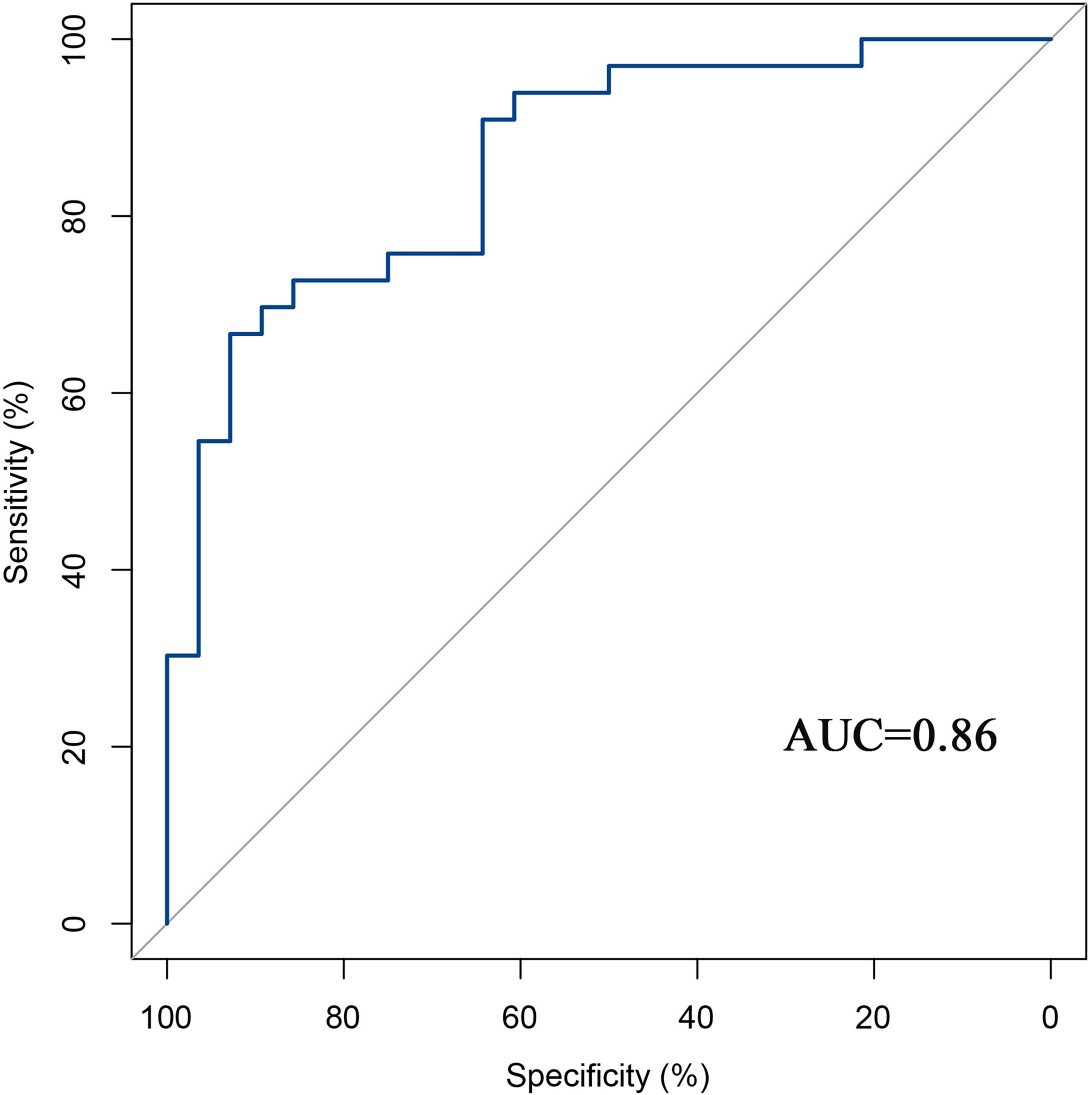
ROC curve analysis for the combined model.

**Figure 9:**
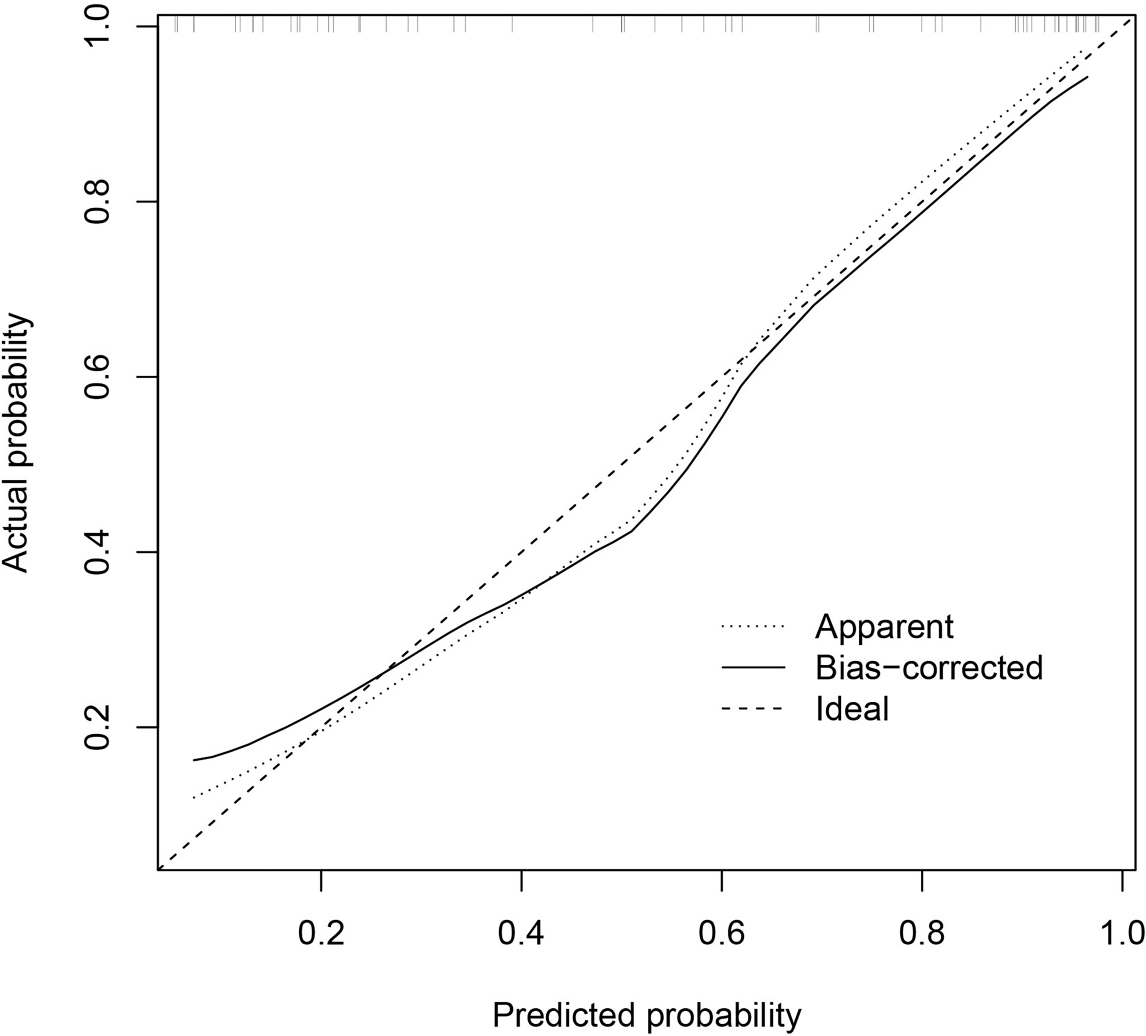
Calibration curve of the combined model indicates a better agreement between the predicted probabilities and the actual observed frequencies.

**Figure 10:**
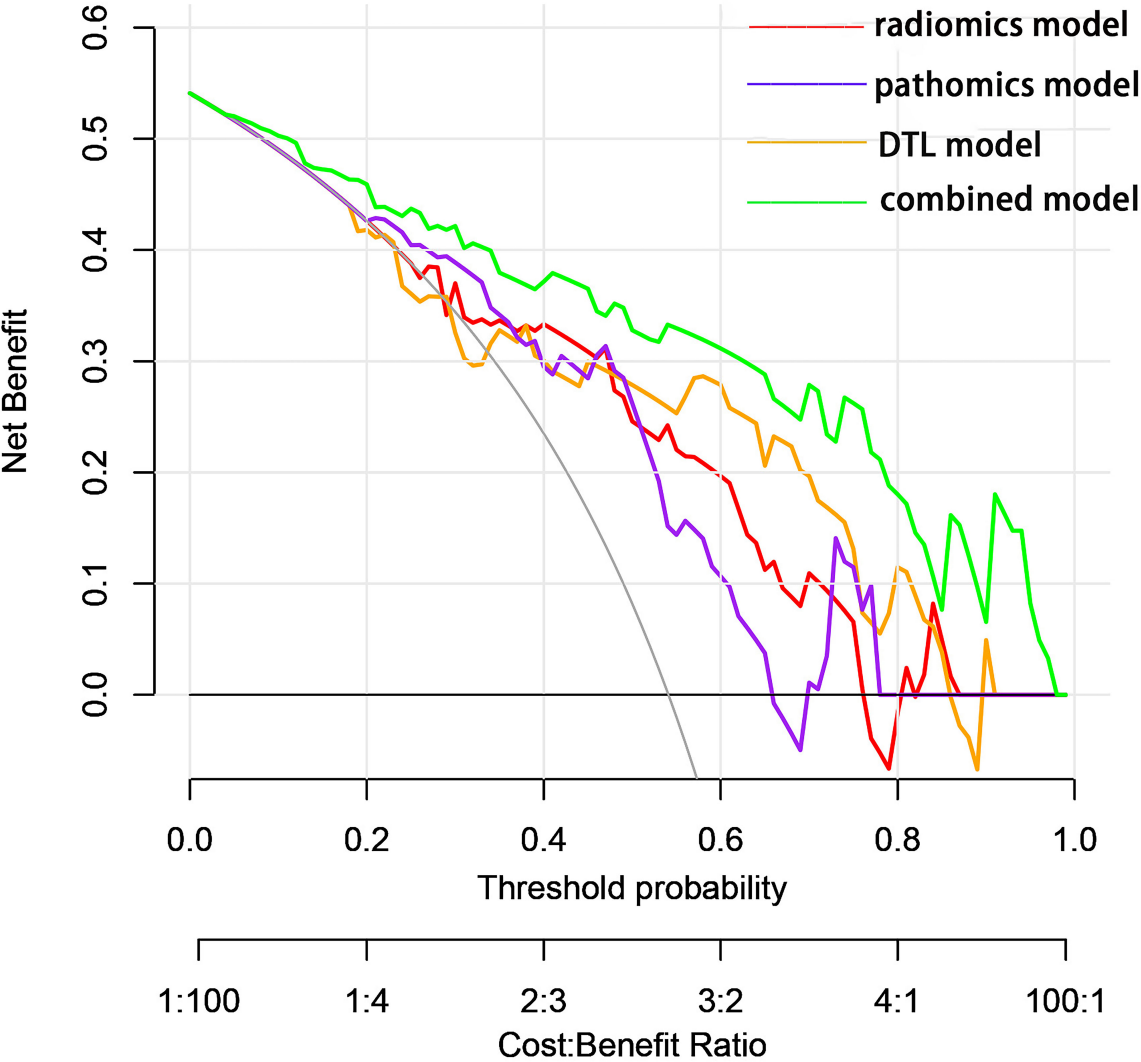
Decision curves showed that each model could achieve clinical benefit and that the net benefit of the combined model was better.

The predictive performance of the models was evaluated using ROC analysis. The best ML model for radiomics was SVM, with an AUC of 0.755 (**Figure 6A**). For DTL and pathomics, the best model was ResNet-50, with AUC values of 0.768, 0.714, 0.684, and 0.752 (**Figures 6B–E**). The nomogram graph showed that DTL contributed the most in the combined model (**Figure 7**), and the AUC of the combined model was 0.86 (**Figure 8**). Calibration curve analysis showed that the joint model has a good fit and strong calibration capability (**Figure 9**). The DCA curve showed that all models had good clinical net benefit, with the combined model showing higher net benefit (**Figure 10**).

### 3.4 Prognosis

**Figure 11:**
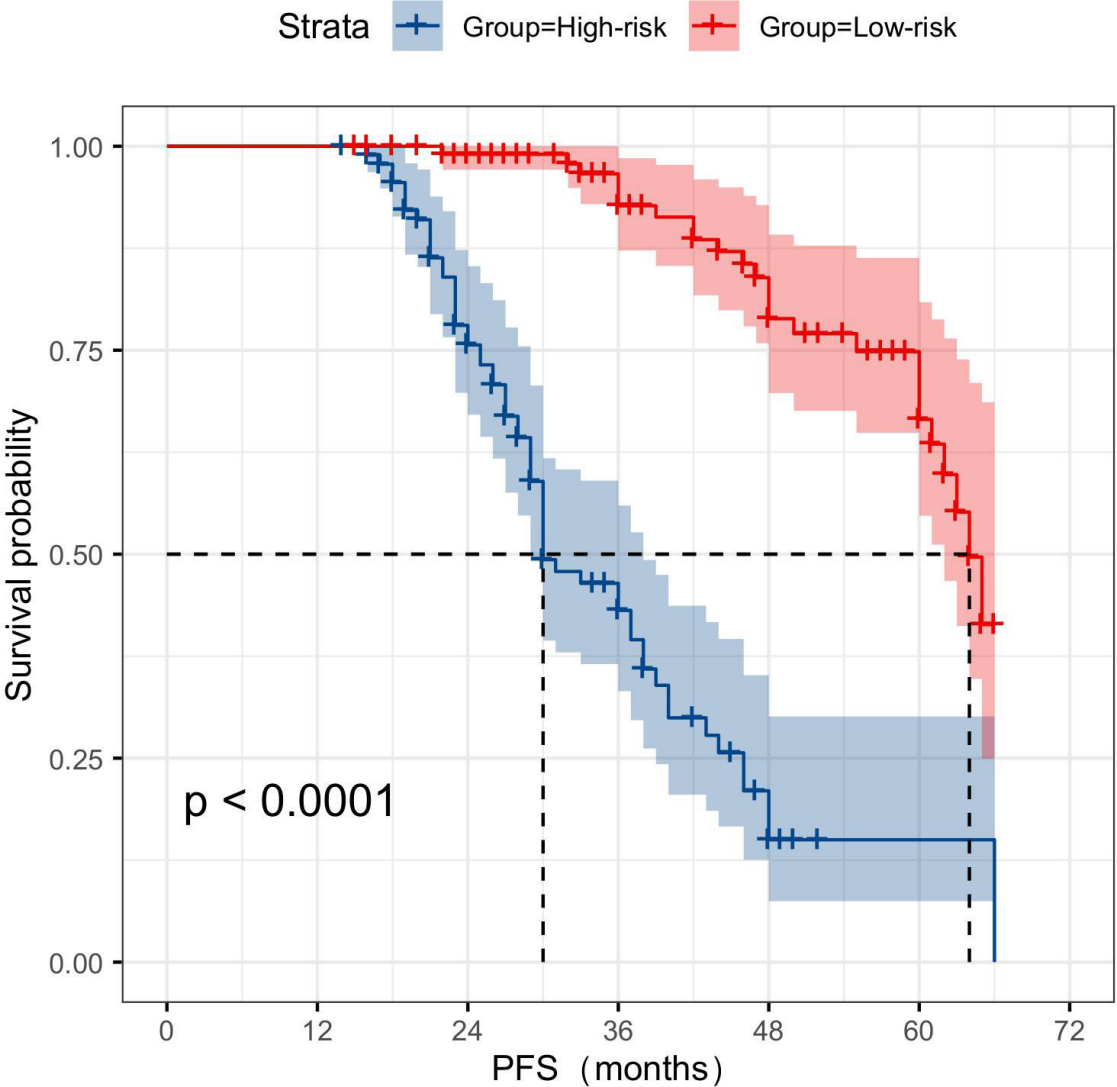
KM survival curve analysis demonstrates that multimodal data can serve as a reliable predictor of the risk of CRPC occurrence. CRPC, castration-resistant prostate cancer.

In the classification study of CRPC risks, a total of 87 patients experienced tumor progression-related events. The KM curve analysis showed that the joint model suggests significantly lower PFS for patients at high risk of CRPC compared to those at low risk (**Figure 11**).

## 4 Discussion

To our knowledge, in this retrospective cohort study conducted across multiple centers, a novel prediction model was developed and validated for the first time. This model integrated radiomics, DTL, and pathomics data to provide strong predictive capabilities in primary prostate cancer progressing to CRPC following two years of ADT. The utilization of multiparametric radiological modeling, as employed in this investigation, may aid urologist in evaluating the probability of CRPC progression and formulating personalized treatment strategies.

The prognosis of CRPC is notably unfavorable, and the challenges in its treatment are diverse among patients (18). The acquisition of reliable data from an initial diagnosis of localized PCa managed with ADT is constrained in clinical practice (19). Previous research has demonstrated a significant correlation between N-glycan score and adverse prognosis in CRPC (20). Additionally, the assessment of skeletal muscle index and skeletal muscle attenuation holds predictive value for the prognosis of metastatic CRPC (21). PSA nadir and Grade 5 were both associated with CRPC progression (22). It was also established that AR-V7 mRNA, significantly predicted biochemical recurrences and CRPC progression (23). However, none of these findings provided specific and prospective indications regarding the likelihood of castration-CRPC progression in patients with PCa. Our approach demonstrated significant predictive performance and provided therapeutic advantage. In addition, the calibration curve and KM survival curve were well-suited for the model and provided useful predictive information for patients with PCa. This finding could potentially be attributed to the multimodal data integration and the selection of suitable AI methodologies.

### 4.1 Multimodal data integration

Data fusion addresses inference problems by amalgamating data from various modalities that provide different viewpoints on a shared phenomenon (24, 25). Consequently, the integration of multiple modalities may facilitate the resolution of such challenges with greater precision compared to the utilization of singular modalities (26). This is particularly important in medicine, as similar results from different measurement techniques might provide different conclusions (27, 28). In recent years, the growing prevalence of original studies utilizing imaging and pathology images in the field of prostate cancer has created an opportunity for AI technology to demonstrate its potential (29, 30). Additionally, DL approaches have direct applications for segmentation, multimodal data integration and model construction (31).

We used late-stage fusion, also known as decision-level fusion, to train a separate model for each modality and then aggregate the predictions from each model to produce a final prediction. Aggregation can be done by averaging, majority voting, and Bayesian-based rules among other methods (32). During the data collection phase, we found that some of the data were missing and incomplete, while late fusion still maintained the predictive power. Since each model is trained individually, aggregation methods, such as majority voting, can be applied even if one mode is missing. In contrast, if the unimodal data do not complement one another or have weak interdependencies, late fusion may be preferred due to its simpler design and fewer parameters in comparison to other fusion procedures. This is also advantageous in instances with insufficient data. In this study, MRI and H&E tissue sections were weakly complementary to each other, and hence our post-fusion model demonstrated good predictive ability. Examples of late fusion include the integration of imaging data with non-imaging inputs, such as the fusion of MRI scans and PSA blood tests for PCa diagnosis (33). Survival prediction using the fusion of genomics and histology profiles by Chen et al. was also performed (34).

### 4.2 Supervised method

In this study, we selected a supervised AI approach for training radiomics models using radiology image annotations with patient outcomes to input data into predefined labels (e.g., cancer/non-cancer) (35). Since the feature extraction was not part of the learning process, the models typically had more simple architecture and lower computation costs. An additional benefit was a high level of interpretability because the predictive features could be related to the data. In contrast, the feature extraction was time-consuming and could translate human bias to the models. Based on the sample size included in this study, the supervised method was sufficient due to its simplicity and ability to learn from our radiomics model.

Self-supervised techniques effectively leverage accessible unlabeled data to acquire superior image features, subsequently transferring this acquired knowledge to supervised models. Consequently, supervised methods like CNNs are employed to address diverse pretexting tasks, wherein labels are automatically generated from the data (36). Notably, self-supervised methods are particularly well-suited for more robust computational systems and higher-resolution images (37, 38).

### 4.3 Model selection for DL

DL is the current state-of-the-art ML algorithm, which simulates the connections between the neurons of the human brain. It learns and extracts complex high-level features from the input data through multi-layer neural networks, thus realizing automatic classification, recognition, and prediction of data. Traditional deep CNNs often encounter the issues of gradient vanishing or gradient explosion as the number of network layers increases, leading to challenging model training. ResNet addresses this problem by introducing the concept of residual connections. The structure promotes the flow of gradients and information transfer, thereby facilitating the training of deeper networks. In this study, we selected DL models including ResNet-50, ResNet-34, ResNet-18, and Vgg19 for pre-training. Comparing these models revealed that ResNet-50 outperformed the others. The main advantage of ResNet-50 lies in its ability to effectively train very deep neural networks while avoiding issues such as gradient vanishing and gradient explosion. Consequently, it excels in image classification tasks and can manage large and complex datasets. Due to its versatile application and remarkable performance, ResNet-50 serves as a benchmark model in various computer vision tasks and is widely utilized in target detection, image segmentation, and image generation. In Lei et al.’s training study of MRI DL involving 396 patients with PCa, training a DL model for PCa classification using pairs of ResNet-50 anti-paradigms improved the generalization and classification abilities of the model (39). In another pathomics study, texture features captured using the ResNet DL framework were able to better distinguish unique Gleason patterns (40).

### 4.4 Limitations

The study has limitations. First, this is a retrospective study from a multicenter institution, and potential biases, such as differences in MRI acquisition parameters, are inevitable. However, as mentioned previously, we completed the data alignment and pre-processed the images to minimize the impact of these differences on the results. Second, key prognostic factors in clinical characterization were not considered in this study due to incomplete clinical data for most patients. Third, our sample size was relatively small, and the number of patients with different Gleason score classifications was unevenly distributed, which may affect the stability and reproducibility of our model. Therefore, the results of this study need to be validated externally using a large sample and a multi-region, multicenter institution in the future.

## 5 Conclusions

In summary, we collected a multimodal dataset from patients who developed CRPC and used it to develop and integrate radiological and histopathological models to improve CRPC risk prediction. This result encourages to conduct further large-scale studies utilizing multimodal DL.

## Data availability statement

The original contributions presented in the study are included in the article/**Supplementary Material**. Further inquiries can be directed to the corresponding authors.

## Ethics statement

The studies involving humans were approved by The Ethics Committee of the Gansu Provincial Geriatrics Association (2022-61). The studies were conducted in accordance with the local legislation and institutional requirements. Written informed consent for participation in this study was provided by the participants’ legal guardians/next of kin. Written informed consent was obtained from the individual(s) for the publication of any potentially identifiable images or data included in this article.

## Author contributions

CZ: Conceptualization, Data curation, Funding acquisition, Investigation, Software, Supervision, Validation, Writing – original draft, Writing – review & editing. Y-FZ: Data curation, Formal analysis, Investigation, Methodology, Software, Validation, Visualization, Writing – original draft. SG: Data curation, Formal analysis, Investigation, Methodology, Software, Writing – review & editing. Y-QH: Data curation, Formal analysis, Investigation, Methodology. X-NQ: Investigation, Methodology, Writing – review & editing. RW: Investigation, Methodology, Writing – review & editing. L-PZ: Investigation, Methodology, Writing – review & editing. D-HC: Resources, Writing – review & editing. L-MZ: Resources, Writing – review & editing. M-XD: Conceptualization, Funding acquisition, Resources, Writing – review & editing, Data curation, Formal analysis, Visualization, Writing – original draft. F-HZ: Conceptualization, Funding acquisition, Resources, Supervision, Writing – review & editing.
