# Supplementary material for: Multimodal data integration for predicting progression risk in castration-resistant prostate cancer using deep learning: a multicenter retrospective study
Source: Front Oncol. 2024 Mar 14;14:1287995. doi: 10.3389/fonc.2024.1287995 (PMC10972942; doi:10.3389/fonc.2024.1287995)
Supplement: Supplementary file 1 [file DataSheet_1.zip › Supplementary materials Table 1.docx]

Supplementary materials Table 1 Main sequence parameters of mp-MRI scans for prostate cancer

| sequence | TR  (ms) | TR(ms) | slice thickness(mm) | Gap | Matrix | NEX | FOV(mm) |
| --- | --- | --- | --- | --- | --- | --- | --- |
| T2WI-FS-TRA | 2800 | 120 | 3 | 0.6 | 256×256 | 2 | 200×200 |
| DWI-ADC-TRA | 4000 | 60 | 3 | 0 | 128×128 | 2 | 200×200 |

Note: TR:repetition time; TE: echo time; TSE: fast self-selected echo; FS: fat suppression sequence;TRA:transverse; T2WI: T2 weighted imaging;DWI: diffusion-weighted imaging; ADC: apparent diffusion coefficient imaging; slice thickness: layer thickness; Gap: layer spacing; Matrix: matrix; NEX: averaged number of times; FOV: field of view.
