# Supplementary material for: Multimodal data integration for predicting progression risk in castration-resistant prostate cancer using deep learning: a multicenter retrospective study
Source: Front Oncol. 2024 Mar 14;14:1287995. doi: 10.3389/fonc.2024.1287995 (PMC10972942; doi:10.3389/fonc.2024.1287995)
Supplement: Supplementary file 1 [file DataSheet_1.zip › Supplementary materials Table 2.docx]

Supplementary materials Table 2. Difference-in-difference analysis of ROI extraction features based on two physicians' labeling

| Features | Radiologist(n=30) | Urologist(n=30) | Z Value | P Value |
| --- | --- | --- | --- | --- |
| ADC-original_firstorder_10Percentile | -0.11(-0.61,0.65) | -0.10(-0.60,0.65) | -0.030 | 0.976 |
| ADC-original_firstorder_Kurtosis | -0.45(-0.65,0.85) | -0.44(-0.66,0.88) | -0.030 | 0.976 |
| ADC-original_firstorder_Median | -0.21(-0.77,0.90) | -0.21(-0.77,0.90) | -0.030 | 0.976 |
| ADC-original_firstorder_Skewness | -0.21(-0.68,0.88) | -0.21(-0.68,0.88) | -0.044 | 0.965 |
| ADC-original_firstorder_TotalEnergy | -0.29(-0.60,0.10) | -0.29(-0.60,0.11) | -0.044 | 0.965 |
| ADC-original_glcm_ClusterShade | -0.08(-0.54,0.77) | -0.08(-0.54,0.77) | -0.015 | 0.988 |
| ADC-original_glcm_Idn | 0.18(-0.48,0.63) | 0.18(-0.47,0.63) | -0.015 | 0.988 |
| ADC-original_glcm_Imc2 | 0.27(-0.07,0.64) | 0.27(-0.06,0.65) | -0.030 | 0.953 |
| ADC-original_glcm_MCC | -0.14(-0.69,0.89) | -0.07(-0.77,0.98) | -0.030 | 0.953 |
| ADC-original_glcm_MaximumProbability | -0.18(-0.75,0.69) | -0.17(-0.78,0.63) | -0.030 | 0.976 |
| ADC-original_glrlm_GrayLevelNonUniformity | -0.31(-0.46,-0.06) | -0.25 (-0.71,0.57) | -0.192 | 0.848 |

We randomly screened 11 features extracted based on ADC sequences and performed Mann-Whitney U test on the two groups of features, and the results showed that there was no statistically significant difference between the two groups of features (p < 0.05).
